# Research progress on abdominal cocoon syndrome

**DOI:** 10.3389/fmed.2026.1767029

**Published:** 2026-02-05

**Authors:** Tao Chen, Aixia Kuang, Jinjian Xiang

**Affiliations:** 1 Department of Gastrointestinal Surgery, The First People’s Hospital of Jingzhou, Jingzhou, Hubei, China; 2 Department of Endocrinology, The First People’s Hospital of Jingzhou, Jingzhou, Hubei, China

**Keywords:** abdominal cocoon, imaging diagnosis, intestinal obstruction, peritoneal fibrosis, surgical treatment, tuberculous peritonitis

## Abstract

Abdominal Cocoon Syndrome (ACS) is an uncommon medical condition defined by the encasement of the small intestine within a robust fibrocollagenous membrane, frequently resulting in intestinal obstruction. The clinical presentation of this syndrome is varied, which complicates its diagnosis due to both its atypical symptoms and infrequency. This review offers an extensive summary of the existing knowledge regarding ACS, concentrating on its causes, clinical manifestations, diagnostic imaging, pathological features, treatment modalities, and prognostic implications. Recent case studies and research have clarified the correlation between ACS and conditions such as tuberculous peritonitis, underscoring the necessity for differential diagnosis. Enhancements in imaging methodologies have facilitated improved preoperative identification, while surgical treatment continues to be the primary approach, with particular attention given to essential surgical techniques and the management of intraoperative complications. Through a systematic evaluation of contemporary literature, this review aspires to elevate clinical awareness, enable prompt diagnosis, and encourage tailored treatment strategies aimed at enhancing patient outcomes in cases of ACS.

## 1 Introduction

ACS is alternatively referred to as encapsulating peritoneal sclerosis (EPS) or sclerosing encapsulating peritonitis (SEP), represents a rare and complex chronic inflammatory disorder. This condition is marked by the development of a robust fibrocollagenous membrane that envelops the small intestine, resulting in varying levels of bowel obstruction (1). The resultant fibrotic encapsulation leads to the constriction of the small bowel within a sac resembling a cocoon, significantly disrupting intestinal motility and functionality (2). The initial accounts of ACS can be traced back to the 20th century, where isolated case reports emphasized its distinctive clinical and intraoperative characteristics. Once deemed an exceedingly rare condition, there has been a notable rise in documented instances in recent years, especially in areas with a high incidence of tuberculosis (TB). Patients generally exhibit symptoms indicative of small bowel obstruction (SBO), including abdominal pain, distension, nausea, vomiting, and constipation; however, these symptoms are prevalent across numerous gastrointestinal ailments, thereby hindering the prompt recognition of the condition (3).

ACS may be caused by many factors, including tuberculosis infection, autoimmune reactions, repeated peritoneal irritation and peritoneal dialysiss (PD) (4). The complex etiology of this syndrome continues to be a subject of ongoing research, with current hypotheses exploring the roles of chronic inflammation, cytokine-driven fibroblast activation, and angiogenesis in the progressive fibrosis and encapsulation that define the condition. The pathophysiological mechanisms associated with ACS are intricate and remain incompletely understood. It is hypothesized that persistent or chronic irritation of the peritoneum—stemming from infectious agents such as *Mycobacterium tuberculosis*, autoimmune responses, or mechanical factors—initiates an inflammatory response within the peritoneal cavity. This inflammatory reaction stimulates the proliferation of fibroblasts and leads to excessive collagen accumulation, resulting in the development of a dense fibrous membrane that encases the intestines. Such encapsulation limits intestinal motility and increases the risk of recurrent or complete SBO in affected individuals (5). Importantly, ACS may also present in idiopathic forms, where no identifiable preceding cause exists, thereby complicating the comprehension of its pathogenesis. Furthermore, various factors, including cytokines and angiogenic mediators, have been recognized as contributors to the fibrotic process, underscoring the complex interactions between immune responses and tissue repair mechanisms in ACS (6).

The diagnosis of ACS presents significant challenges, primarily due to its nonspecific clinical manifestations and the absence of distinctive signs during standard diagnostic evaluations. Imaging techniques are crucial for preoperative identification, with contrast-enhanced computed tomography (CT) emerging as the most informative imaging modality. Typically, CT findings illustrate a mass of small bowel loops surrounded by a thick fibrous membrane, frequently associated with loculated ascites or peritoneal thickening. Although these imaging characteristics are indicative of ACS, they are not exclusive and can resemble other causes of intestinal obstruction, such as internal hernias or adhesions. As a result, numerous cases are only conclusively identified during surgical procedures, either exploratory laparotomy or laparoscopy, where the defining fibrous cocoon encasing the intestines can be directly observed (7). This intraoperative identification not only affirms the diagnosis but also informs immediate surgical intervention.

From a therapeutic standpoint, the management of ACS primarily necessitates surgical intervention, particularly for patients who exhibit acute or recurrent bowel obstructions (8). The conventional surgical technique entails careful adhesiolysis accompanied by the thorough excision of the fibrous membrane that encases the bowel, thereby liberating the entrapped intestinal loops. Typically, bowel resection is avoided unless there is clear evidence of nonviable intestinal tissue, as such procedures can lead to increased morbidity. Surgical procedures are inherently risky due to the presence of dense adhesions and the fragility of the intestinal tissue, with patient outcomes being influenced by the timing of the surgery and the severity of the disease. In certain instances, particularly those involving patients with mild symptoms or those deemed unfit for surgical intervention, conservative management strategies—including nutritional support and symptomatic relief—may be appropriate. Nonetheless, prompt identification and timely surgical intervention are paramount in enhancing prognosis and mitigating the complications associated with prolonged obstruction and ischemia (9).

Given the infrequency and intricacy of ACS, it is imperative to conduct a thorough review of contemporary literature to amalgamate existing knowledge regarding its causes, diagnostic techniques, and treatment approaches (10). A comprehensive understanding of the multifactorial origins, the ability to identify subtle clinical and radiological indicators, and the development of personalized management strategies are crucial for enhancing patient outcomes. This review seeks to integrate the most recent research outcomes and clinical insights to furnish healthcare professionals with an updated framework for the diagnosis and management of this complex condition, particularly in endemic areas where tuberculosis and other contributing factors may increase the likelihood of its occurrence. By improving awareness and promoting prompt diagnosis, the complications associated with abdominal cocoon syndrome can be alleviated, thereby ultimately enhancing the quality of life for patients (11, 12).

## 2 Etiology and pathogenesis of abdominal cocoon syndrome

### 2.1 Relationship between tuberculous peritonitis and abdominal cocoon syndrome

Tuberculous peritonitis serves as a notable etiological contributor to the emergence of ACS, which is also referred to as encapsulating peritoneal sclerosis or sclerosing peritonitis. This condition manifests when chronic inflammation, instigated by an infection with *Mycobacterium tuberculosis*, induces a fibrotic response within the peritoneum. This process results in the formation of a dense fibrous membrane that envelops the small intestine, effectively creating a “cocoon” around it (13). The underlying pathophysiology entails a prolonged tuberculous infection that incites ongoing peritoneal inflammation, which in turn promotes fibroblast proliferation and collagen accumulation. This cascade ultimately leads to significant peritoneal fibrosis and the formation of adhesions, which impede intestinal motility and precipitate mechanical obstruction (14). In clinical practice, individuals suffering from tuberculous abdominal cocoon frequently exhibit nonspecific symptoms, including abdominal pain, distension, and signs indicative of subacute or acute intestinal obstruction. Additionally, the presence of ascites and thickening of the peritoneum are typical yet nonspecific findings that may complicate the differential diagnosis, often resulting in misdiagnosis as various other intra-abdominal conditions, such as carcinomatosis or alternative causes of peritonitis (15).

The diagnostic complexities associated with tuberculous ACS are significant, primarily due to the absence of distinctive clinical indicators and the nonspecific characteristics of imaging results. CT is regarded as the definitive imaging technique, as it can display typical manifestations such as clusters of small bowel loops surrounded by a thickened peritoneal membrane, enhancement of the peritoneum, and the presence of loculated ascites (16). Nevertheless, these imaging features can also be present in various other peritoneal conditions, making it essential to obtain histopathological evidence through surgical biopsy or analysis of peritoneal fluid to confirm the diagnosis unequivocally (17). Both laparoscopy and laparotomy not only enable direct observation of the fibrous cocoon but also assist in adhesiolysis and the removal of the fibrotic membrane, which are vital for alleviating symptoms and restoring normal bowel function.

From a therapeutic perspective, the fundamental approach to managing tuberculous abdominal cocoon encompasses a dual strategy involving extended anti-tuberculous chemotherapy alongside surgical procedures. The primary objective of anti-tuberculous therapy is to address the root infection and mitigate the inflammatory response, whereas surgical intervention is indicated for patients experiencing complications, including bowel obstruction, perforation, or inadequate response to medical treatment. Surgical management generally entails meticulous excision of the fibrous capsule and adhesiolysis to liberate the entrapped bowel segments. Nonetheless, achieving a preoperative diagnosis poses significant challenges, often resulting in surgeries being conducted either on an urgent basis or after considerable delays, which may elevate the risk of morbidity (18). The postoperative prognosis is influenced by the severity of the disease and the timing of the intervention; thus, prompt identification coupled with an integrated medical-surgical approach tends to yield the most favorable outcomes. In summary, tuberculous peritonitis is a significant yet frequently overlooked contributor to abdominal cocoon syndrome, highlighting the necessity for increased clinical vigilance, enhanced diagnostic methodologies, and a multidisciplinary approach in order to optimize patient outcomes.

### 2.2 Pathogenesis of spontaneous (idiopathic) abdominal cocoon syndrome

Spontaneous or idiopathic ACS is defined by the development of a robust fibrocollagenous membrane that envelops the small intestine, resulting in varying levels of intestinal obstruction. In contrast to secondary forms that are linked to identifiable etiologies such as PD or tuberculosis, idiopathic ACS arises in the absence of any discernible triggering factors, rendering its pathogenesis enigmatic. Existing data indicate that the fundamental mechanism may involve an abnormal immune response coupled with localized, excessive inflammation of the peritoneum. It is proposed that subclinical or chronic low-grade peritoneal inflammation may instigate the activation of peritoneal fibroblasts. These fibroblasts subsequently proliferate and synthesize an excess of collagen, culminating in progressive fibrosis and encapsulation of the intestinal loops (19). Histopathological analyses of surgically removed membranes typically demonstrate fibro-collagenous tissue accompanied by mild chronic inflammatory infiltrates, thereby corroborating the involvement of immune-mediated fibrogenesis (20). The fibrotic process is characterized by the activation of fibroblasts and the deposition of extracellular matrix components, particularly collagen, which contributes to the thickening of the peritoneal membrane and results in the distinctive cocoon-like encapsulation.

Despite the absence of a clearly defined cause for idiopathic ACS, certain clinical observations suggest that previous abdominal traumas, such as surgical interventions or PD, might serve as potential triggers or aggravators for the fibrotic process, even in the absence of a clear secondary etiology (21). These elements could provoke localized irritation and inflammation within the peritoneum, thereby initiating a sequence of fibroblast activation and collagen accumulation. Nevertheless, idiopathic instances frequently arise without a documented history of these risk factors, implying that additional, yet-to-be-identified immunological or environmental variables might play a role in the onset of the disease. The higher incidence of idiopathic ACS in tropical and subtropical regions suggests the possibility of geographical or genetic factors that may influence immune responses (22).

In clinical settings, idiopathic ACS is characterized by recurrent instances of SBO, abdominal discomfort, and, in certain cases, the presence of palpable masses within the abdomen (23). Imaging techniques, particularly contrast-enhanced CT, are essential for preoperative diagnosis, as they can illustrate small bowel loops that are grouped together and enveloped by a thickened membrane, often referred to as a “cocoon” (24). Despite the advancements in imaging technology, a conclusive diagnosis is often made during intraoperative procedures, specifically during exploratory laparotomy, where the fibrous membrane becomes visible and is subsequently excised (25, 26). The standard treatment approach consists of surgical adhesiolysis and membrane resection, which aims to alleviate the encapsulation of the bowel and restore normal intestinal function while minimizing the need for bowel resection (11, 23).

In summary, the development of spontaneous abdominal cocoon syndrome is characterized by a multifaceted interaction between immune system dysregulation and the deposition of collagen mediated by fibroblasts, leading to an advancement of peritoneal fibrosis and encapsulation of the intestines (27). Although the precise initiating factors are not well understood, the fibrotic mechanism plays a pivotal role in the progression of the disease, necessitating surgical procedures to relieve obstructions and avert further complications. Additional investigations are essential to clarify the molecular mechanisms underlying fibrosis and to discover possible preventive or therapeutic strategies for this uncommon yet complex condition.

### 2.3 Other etiologies and related factors

ACS is an uncommon medical condition marked by the development of a robust fibrocollagenous membrane that envelops the small intestine, resulting in varying levels of intestinal obstruction (19). Although the primary cause remains largely unknown in numerous instances, multiple secondary factors and associated causes have been recognized, with patients undergoing PD identified as a notably high-risk population. Prolonged exposure toPD solutions, which contain glucose degradation products that provoke peritoneal inflammation, fibrosis, and sclerosis, is widely acknowledged as a significant predisposing factor for ACS. This persistent inflammatory stimulus contributes to gradual peritoneal damage, ultimately resulting in the formation of a fibrous cocoon surrounding the intestinal loops (28). Clinical observations indicate that the prevalence of ACS escalates with the duration of PD, particularly after 5 years, with recurrent peritonitis episodes further intensifying peritoneal injury and fibrosis (29, 30). Furthermore, patients suffering from systemic autoimmune disorders, such as systemic lupus erythematosus (SLE), who are on PD may exhibit an increased risk due to immune-mediated peritoneal inflammation, as evidenced by cases where dysregulation of the immune system associated with SLE plays a role in the onset of ACS (31). In these individuals, the clinical trajectory may be complicated by severe intestinal obstruction and systemic complications, thereby necessitating a comprehensive, multidisciplinary approach to treatment (32).

In addition to PD, a prior history of abdominal surgical procedures is acknowledged as a significant risk factor for ACS. Such surgical interventions can lead to peritoneal damage and the formation of adhesions, which may subsequently progress to fibrotic encapsulation in individuals who are predisposed to this condition (19). Instances of ACS occurring post-laparoscopic cholecystectomy and other abdominal surgeries have been documented, emphasizing the influence of surgical trauma and postoperative inflammatory responses on the development of ACS (33, 34). Moreover, infectious agents, including tuberculosis and recurrent bacterial peritonitis, have been recognized as possible catalysts for peritoneal fibrosis and the formation of a cocoon-like structure. Nevertheless, in numerous cases, thorough investigations do not reveal a clear infectious origin, highlighting the intricate nature of this disease (35). Additionally, adverse drug reactions and exposure to specific pharmaceuticals may play a role in inducing peritoneal inflammation and fibrosis, although the literature offers less clarity regarding these associations.

Instances of ACS occurring alongside congenital or developmental anomalies are infrequently documented, including cases involving supernumerary remnants of the Wolffian duct, which may offer novel perspectives on the embryological determinants affecting peritoneal pathology (35). Furthermore, there have been reports of ACS coexisting with various intestinal deformities, intra-abdominal neoplasms, or autoimmune disorders beyond systemic lupus erythematosus (SLE), indicating that a multifactorial origin may be present in certain individuals (36). For example, IgG4-related sclerosing mesenteritis, a chronic inflammatory disorder marked by lymphoplasmacytic infiltration and fibrotic changes, has recently been identified as a potential contributor to the formation of abdominal cocoon, thereby broadening the range of immune-mediated peritoneal diseases that can result in ACS (37).

The etiology of ACS is varied, involving chronic irritation of the peritoneum due to PD, surgical trauma, infections, autoimmune disorders, and infrequent congenital anomalies (38). Identifying these various causative factors is essential for prompt diagnosis and individualized management. For patients undergoing PD, careful surveillance for indications of peritoneal sclerosis, coupled with timely interventions, can avert the escalation to severe bowel obstruction (39). Likewise, a comprehensive medical and surgical history may assist healthcare providers in considering abdominal cocoon syndrome in patients who present with unexplained intestinal obstruction. Additional investigations are necessary to clarify the pathophysiological mechanisms that connect these factors and to refine both preventive and therapeutic approaches for this complex condition.

## 3 Clinical manifestations and diagnostic challenges

### 3.1 Typical clinical manifestations

ACS manifests through a range of clinical symptoms predominantly associated with recurrent SBO episodes. The primary indicators of this condition encompass intermittent or continuous abdominal pain, abdominal distension, nausea, vomiting, and difficulties in defecation, all of which signify the mechanical disruption of intestinal transit due to the fibrous encapsulation surrounding the bowel loops. Patients frequently experience colicky abdominal pain, which may be accompanied by nausea and vomiting that can either be bilious or non-bilious, contingent upon the severity of the obstruction. Abdominal distension is commonly noted as a result of bowel dilation occurring proximal to the obstruction site. Furthermore, constipation or obstipation may arise, reflecting compromised bowel motility. These clinical manifestations can present in an episodic manner, with some cases resolving spontaneously, while others may progressively deteriorate, culminating in acute intestinal obstruction that necessitates urgent medical intervention (40, 41). The clinical presentation is often nonspecific, complicating the diagnostic process; thus, patients may initially be misdiagnosed with more prevalent causes of bowel obstruction, such as adhesions or internal hernias. Imaging techniques, particularly contrast-enhanced CT, are instrumental in revealing characteristic features, including clusters of small bowel loops enveloped by a thick fibrous membrane, which are sometimes referred to as the “cocoon” or “cauliflower” sign, aiding in the differentiation of abdominal cocoon syndrome from other underlying causes (42).

It is important to note that a portion of patients may exhibit no symptoms or only mild and nonspecific manifestations. In such instances, abdominal cocoon syndrome is frequently identified incidentally during laparotomy or laparoscopy conducted for other reasons, such as the assessment of unexplained abdominal discomfort or during surgical procedures for bowel obstruction of uncertain origin (43, 44). The discovery of a fibrous membrane enveloping the small intestine in the absence of significant clinical symptoms highlights the diverse clinical presentation associated with this condition (45). Some individuals may carry the disease for prolonged durations without experiencing notable symptoms, whereas others may encounter recurrent or acute obstruction episodes (46). This variability could be influenced by factors such as the extent of membrane development, the degree of bowel encapsulation, and the presence of adhesions.

In instances of severe or advanced conditions, individuals may exhibit acute symptoms indicative of complete bowel obstruction, which include intense abdominal pain, vomiting, abdominal swelling, and a state of absolute constipation. Although infrequent, serious complications such as bowel ischemia, necrosis, or perforation can arise, resulting in septic manifestations that necessitate urgent surgical intervention (47, 48). The acute presentation may resemble other surgical emergencies, including strangulated internal hernias or perforated appendicitis, thereby complicating the preoperative diagnostic process. It is crucial to maintain a high level of suspicion, particularly in patients with no history of abdominal surgeries who display symptoms of bowel obstruction along with imaging results that imply bowel encapsulation (49).

In conclusion, the characteristic clinical presentations associated with ACS primarily involve recurrent or persistent SBO symptoms, such as abdominal discomfort, swelling, nausea, vomiting, and constipation (41). Nevertheless, the clinical manifestations can vary significantly, ranging from asymptomatic incidental discoveries to urgent surgical crises. Prompt identification of these symptoms, along with relevant radiological evidence, is crucial for facilitating an accurate diagnosis and effective management, ultimately enhancing patient prognosis (40).

### 3.2 Atypical presentations and risks of misdiagnosis

ACS commonly referred to as abdominal cocoon, predominantly presents as chronic or recurrent SBO. Nonetheless, atypical manifestations may hinder prompt diagnosis and elevate the risk of erroneous identification. A small fraction of cases may exhibit subacute intestinal obstruction or may be complicated by intestinal perforation, which could resemble other acute abdominal conditions. For example, a documented case of idiopathic ACS involved a 67-year-old male who exhibited symptoms such as constipation, vomiting, and abdominal discomfort—symptoms that, while prevalent, are nonspecific and can readily be linked to various other gastrointestinal issues (50). Although imaging studies indicated ileal thickening, a definitive diagnosis was only made during surgery, underscoring the diagnostic difficulties associated with atypical presentations (20). These subacute or acute symptoms can create confusion with more prevalent causes of bowel obstruction, such as postoperative adhesions, intestinal tuberculosis, or neoplasms. Although intestinal perforation is infrequent, it further complicates the clinical scenario by mimicking peritonitis due to other causes, which may result in delays in necessary surgical treatment.

Misdiagnosis poses a considerable challenge in the context of ACS owing to its infrequent occurrence and the nonspecific nature of its clinical and radiological manifestations. This condition is often confused with intestinal adhesions, tuberculosis, or neoplasms, all of which share similar symptoms, including abdominal discomfort, distension, and obstructive indicators. For instance, in a case involving a young female patient who experienced recurrent episodes of intestinal obstruction and had a background of abdominal trauma and surgical interventions, the diagnosis of an abdominal cocoon was ultimately established only after several surgical explorations uncovered significant fibrous encapsulation and adhesions (12). This scenario highlights the diagnostic challenges, as the initial symptoms were misattributed to postoperative complications and a pelvic abscess, resulting in a delayed identification of ACS (51). Imaging techniques, particularly contrast-enhanced CT, play a crucial role in the preoperative assessment, as they can identify distinctive features such as small bowel loops clustered and surrounded by a thick fibrous membrane. However, a conclusive diagnosis frequently necessitates intraoperative investigation, particularly in atypical presentations.

Delays or inaccuracies in diagnosis can lead to significant repercussions, such as extended obstruction, bowel ischemia, and perforation, all of which contribute to increased morbidity and mortality rates. The elevated mortality rate associated with SBO underscores the necessity for enhanced clinical awareness, particularly in individuals presenting with recurrent or unexplained bowel obstructions lacking a definitive cause (52). Timely identification, achieved through a blend of clinical attentiveness and suitable imaging techniques, can enable prompt surgical intervention, which remains the primary approach for managing symptomatic cases (53). Surgical procedures, including the excision of the fibrous cocoon and adhesiolysis, can alleviate obstruction and avert further complications. In cases that are asymptomatic or exhibit mild symptoms, conservative management may be an option; however, it necessitates meticulous observation to prevent the escalation to acute clinical scenarios (20).

In conclusion, atypical manifestations of SBO, including subacute obstruction or intestinal perforation, present considerable diagnostic difficulties and heighten the likelihood of misdiagnosing these conditions as more prevalent abdominal disorders such as adhesions, tuberculosis, or neoplasms (25). A heightened awareness of these unusual presentations, along with the utilization of advanced imaging modalities and a strong clinical suspicion, is essential to prevent diagnostic delays and enhance patient outcomes. Once a diagnosis is confirmed, surgical intervention remains the fundamental approach to treatment, highlighting the necessity for prompt and precise recognition of this uncommon yet potentially life-threatening condition.

### 3.3 Clinical significance of diagnosis

The prompt and precise identification of ACS is of paramount clinical importance, primarily because it aids in circumventing unnecessary surgical procedures and their associated complications. ACS is an uncommon etiological factor for SBO, characterized by the encasement of the intestines within a robust fibrocollagenous membrane. This condition often leads to recurrent episodes of intestinal obstruction, manifesting with nonspecific symptoms, including abdominal pain, distension, nausea, and vomiting (54, 55). Given its infrequency and the vague nature of its clinical presentation, ACS is frequently misdiagnosed or only recognized during surgical intervention, which can lead to inappropriate or delayed therapeutic measures (17). The early detection of ACS, particularly through imaging techniques such as contrast-enhanced CT, is crucial, as CT can unveil distinctive features such as clustered, matted small bowel loops enveloped by a thickened membrane. This information is invaluable for preoperative diagnosis and aids in surgical planning. Such preoperative insights enable surgeons to customize their surgical strategies, thereby minimizing the extent of resections and concentrating on membrane excision and adhesiolysis, which are essential components of treatment (55). Furthermore, timely diagnosis diminishes the likelihood of complications, including bowel ischemia, perforation, and malnutrition, which may arise from prolonged obstruction or unsuitable surgical management (56, 57).

A comprehensive clinical history is crucial for raising the suspicion of ACS, particularly in cases where patients exhibit recurrent or chronic intestinal obstruction without an evident etiology. Key historical indicators include a past infection with tuberculosis, a history of undergoing PD, and previous abdominal surgical interventions, all of which are acknowledged risk factors for secondary types of ACS (44). The surgical background, particularly involving abdominal injuries or multiple laparotomies, warrants careful inquiry, as it may contribute to the development of peritoneal fibrosis and the formation of a cocoon (19). In certain idiopathic instances, identifiable predisposing factors may be absent, highlighting the necessity for clinicians to maintain a heightened level of suspicion when confronted with unexplained bowel obstruction (23, 58).

In conclusion, the early identification of ACS holds considerable clinical importance as it helps avert unnecessary extensive surgical procedures and the potential complications that accompany them, thereby allowing for more focused surgical interventions. It is crucial to identify significant risk factors, including tuberculosis, PD, and previous abdominal surgeries, through comprehensive patient history assessments, which can lead to the timely consideration of ACS in the differential diagnosis (59). Furthermore, augmenting clinical suspicion with advanced imaging modalities, particularly CT, significantly improves diagnostic precision and enhances patient outcomes by enabling prompt and suitable management strategies (55).

## 4 Advances in imaging diagnosis

### 4.1 Diagnostic value of computed tomography (CT)

**Figure 1:**
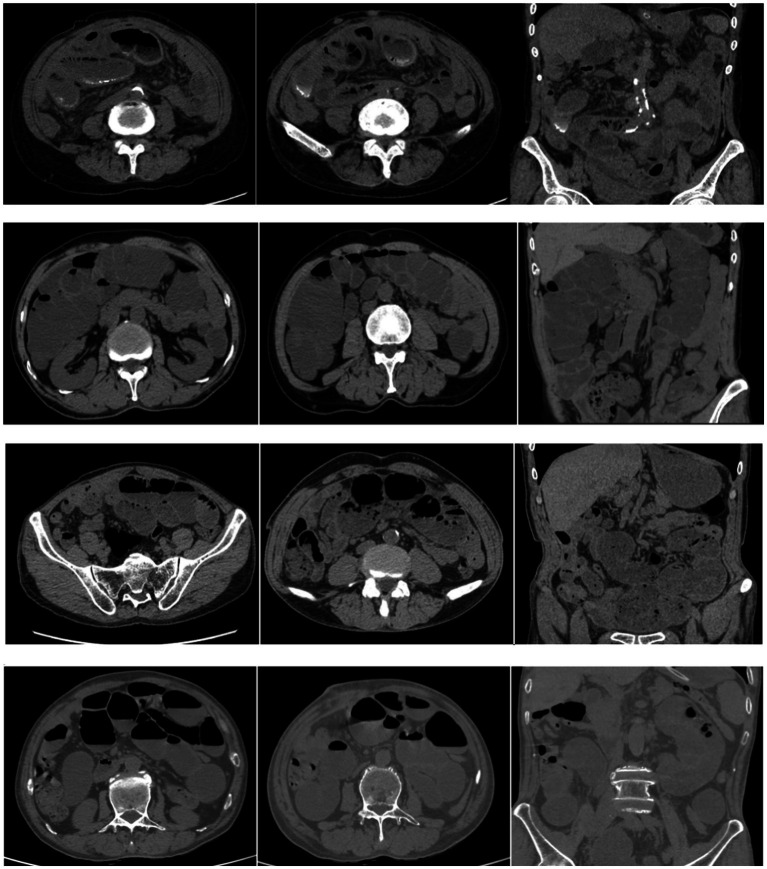
CT images of patients with abdominal cocoon syndrome.

CT is essential in the preoperative identification of ACS. A defining feature observed in CT imaging is the clustering of small bowel loops that are enveloped by a robust, thick fibrous membrane, often likened to a “cocoon” or “capsule” (27). This encapsulation results in distinctive imaging characteristics, such as concentrated and fixed intestinal loops surrounded by a dense fibro-collagenous sac, frequently accompanied by indications of peritoneal thickening and, in certain instances, calcifications present within the membrane (60), We have listed some preoperative CT images of patients with abdominal cocoon syndrome, presenting some of the above-mentioned features, as shown in Figure 1. These observations illustrate the underlying pathological mechanism involving the proliferation of fibro-collagenous tissue and peritoneal fibrosis that encases the small intestine, leading to varying levels of bowel obstruction and ischemia. Numerous case reports and series have illustrated that CT imaging typically reveals a sac-like formation surrounding the small bowel loops, often lacking a discernible transition point in obstruction cases, and occasionally accompanied by mesenteric congestion or ascites, which may suggest ischemia or inflammation (61). The characteristic “cocoon” or “capsule” appearance on CT is strongly indicative of ACS, assisting clinicians in distinguishing it from alternative causes of SBO, such as adhesions, hernias, or neoplasms. The identification of peritoneal thickening and calcifications further corroborates the diagnosis, particularly in secondary forms of ACS associated with PD or tuberculosis (62). Notably, CT not only aids in diagnosis but also plays a crucial role in surgical planning by outlining the extent of bowel involvement and the fibrous membrane, thereby guiding the surgeon in procedures such as adhesiolysis and membrane excision (1). Although ACS is infrequently encountered and presents with nonspecific clinical symptoms, CT has established itself as the gold standard imaging technique, facilitating preoperative detection in a considerable number of cases, which is vital for timely surgical intervention and enhanced patient outcomes (63–65). In conclusion, the diagnostic significance of CT lies in its capacity to visualize the distinctive encapsulation of the small bowel within a dense fibrous membrane, known as the “cocoon sign,” along with associated peritoneal alterations, collectively providing a reliable and non-invasive approach for diagnosing ACS prior to surgical intervention.

### 4.2 The auxiliary role of magnetic resonance imaging (MRI) and ultrasound

MRI and ultrasound have become significant supplementary methods in the diagnosis and treatment of ACS, enhancing the capabilities of other imaging techniques such as CT. MRI possesses unique benefits in depicting the fibrotic alterations typical of ACS, especially highlighting the thickened peritoneal membrane that surrounds the bowel loops and the limited mobility of the intestinal segments ensconced within this fibrous structure (66). In contrast to CT, MRI is devoid of ionizing radiation, rendering it a safer alternative for repeated assessments, particularly for younger or more susceptible patients. The exceptional soft-tissue contrast resolution inherent to MRI facilitates precise identification of the fibrous membrane and evaluation of the degree of bowel involvement, which is essential for effective preoperative planning. MRI can reveal the distinctive encapsulation of the small intestine by a low-signal-intensity membrane on both T1- and T2-weighted images, alongside indications of bowel loop aggregation and diminished peristalsis, thereby offering a non-invasive approach to corroborate the diagnosis of ACS prior to surgical intervention. This imaging technique proves especially beneficial in scenarios where CT results are ambiguous or when there is a concern regarding radiation exposure (67).

Ultrasound functions as a swift, bedside imaging modality that proves particularly advantageous in emergency situations (68). It is capable of rapidly identifying characteristic features of abdominal cocoon, including clusters of bowel loops surrounded by a hyperechoic fibrous membrane, as well as loculated fluid collections in the peritoneal cavity. Due to its portability and absence of ionizing radiation, ultrasound is considered an optimal initial imaging technique in acute scenarios, such as intestinal obstruction or acute abdominal conditions, where timely diagnosis is critical. This imaging approach can also demonstrate limited bowel motility and encapsulation, which assists in differentiating abdominal compartment syndrome from other etiologies of bowel obstruction (48). Additionally, Doppler ultrasound may facilitate the assessment of bowel vascularity, which is crucial for detecting ischemic alterations resulting from strangulation within the cocoon. Despite its dependence on the operator’s skill and limitations imposed by patient physique and bowel gas, ultrasound remains an indispensable complementary tool, particularly in situations necessitating immediate imaging when more advanced techniques are unavailable (69).

The utilization of both MRI and ultrasound significantly improves the diagnostic precision for abdominal cocoon syndrome by offering comprehensive visualization of the fibrotic alterations in the peritoneum and the encapsulation of the bowel, all while avoiding radiation exposure. This synergistic approach aids in achieving an earlier diagnosis and ensures prompt surgical intervention. Furthermore, their concurrent application can enhance patient outcomes by minimizing diagnostic delays and assisting in surgical planning, especially in intricate or atypical scenarios where there is a strong clinical suspicion, yet conventional imaging yields inconclusive results.

## 5 Pathological features and molecular mechanisms

### 5.1 Histological features

The histological features associated with ACS are primarily characterized by a fibrous capsule predominantly consisting of collagen fibers and fibroblasts. This capsule envelops the loops of the small intestine, leading to the distinctive cocoon-like formation. The presence of this fibrocollagenous membrane is a defining characteristic of ACS and signifies a persistent fibrotic process occurring within the peritoneal cavity. Histopathological analysis generally demonstrates a dense network of fibrous tissue interspersed with spindle-shaped fibroblasts, which play a crucial role in collagen synthesis and deposition, thereby enhancing the stability of the encapsulating membrane. Additionally, this fibrotic reaction is frequently accompanied by the infiltration of chronic inflammatory cells, such as lymphocytes and plasma cells, which suggest an ongoing or prior inflammatory trigger that contributes to the fibrogenic process. The presence of this chronic inflammatory environment implies that immune-mediated mechanisms may be significantly involved in the development of the fibrous encapsulation observed in ACS (35, 70).

In certain instances, the histological characteristics surpass a mere fibrotic and lymphoplasmacytic infiltrate, incorporating eosinophilic infiltration, which indicates a potential immunological aspect in the disease mechanism. The detection of eosinophils within the fibrous capsule implies that hypersensitivity or allergic-type immune reactions might be involved in the onset or advancement of the abdominal cocoon. Notably, this eosinophilic infiltration is not consistently observed; however, it has been recorded in specific cases, emphasizing the variability of the immune response in ACS. Additionally, some studies report the presence of IgG4-positive plasma cell infiltration in the fibrous tissue, which corresponds with the notion of IgG4-related sclerosing mesenteritis presenting as an abdominal cocoon. This particular subtype is distinguished by a pronounced lymphoplasmacytic infiltrate abundant in IgG4-positive plasma cells, storiform fibrosis, and obliterative phlebitis, thus revealing a unique immunopathological mechanism that underlies the fibrotic encapsulation (37).

The observed variability in histological characteristics, encompassing the extent of fibrosis, the composition of the inflammatory infiltrate, and the presence or absence of particular subsets of immune cells, such as eosinophils and IgG4-positive plasma cells, indicates that ACS represents a diverse condition with various potential origins that ultimately lead to a shared pathway of peritoneal fibrosis and encapsulation. Immunohistochemical analyses play a crucial role in distinguishing ACS from other causes of peritoneal fibrosis and in uncovering underlying conditions, including IgG4-related disease or remnants of embryonic structures, which are notably observed in uncommon pediatric instances involving supernumerary Wolffian duct derivatives (35, 37).

The histological characteristics associated with abdominal cocoon syndrome are primarily characterized by the presence of a fibrous collagenous membrane, which results from the activity of fibroblasts and subsequent collagen accumulation. This structural alteration is often accompanied by a chronic inflammatory infiltrate that may consist of lymphocytes, plasma cells, and, on rare occasions, eosinophils. The histopathological features not only substantiate the diagnosis but also offer valuable insights into the potential immunological and fibrogenic processes that may be responsible for the disease’s progression. Such understanding could have significant implications for the development of targeted therapeutic strategies in the future (70, 71).

### 5.2 Molecular and cellular mechanism studies

The molecular and cellular mechanisms associated with ACS, predominantly involve inflammatory pathways that facilitate fibrosis. A crucial inflammatory mediator in this process is transforming growth factor-beta (TGF-*β*), which is integral to the fibrotic progression characteristic of ACS. TGF-*β* is a versatile cytokine that modulates the production and deposition of the extracellular matrix (ECM), thereby promoting the proliferation of fibroblasts and their differentiation into myofibroblasts—key effector cells responsible for collagen synthesis and the formation of fibrotic tissue. In the case of ACS, chronic peritoneal inflammation, whether idiopathic or triggered by factors such as infections, PD, or autoimmune disorders, results in the persistent upregulation of TGF-*β* signaling. This upregulation leads to excessive accumulation of ECM, culminating in the development of a thick fibrocollagenous membrane that encases the loops of the small intestine, consequently causing mechanical obstruction and hindering intestinal motility (65, 72). Histopathological analyses of excised membranes from patients with ACS consistently demonstrate significant fibrosis characterized by a high density of collagen fibers, which is associated with increased TGF-*β* expression within the peritoneal tissue. Furthermore, TGF-β promotes epithelial-to-mesenchymal transition (EMT) in mesothelial cells that line the peritoneum, which further enhances the proliferation of fibrogenic cell populations and sustains the fibrotic process. This cascade of fibrosis is not only central to the pathophysiology of ACS but also represents a potential therapeutic target for halting or reversing the progression of the disease. A thorough understanding of TGF-*β*’s role in ACS offers valuable insights into the molecular mechanisms driving peritoneal fibrosis and highlights the necessity of managing inflammatory stimuli to avert the onset or aggravation of ACS.

The development of the abdominal cocoon is characterized by intricate interactions between the activation of immune cells and the remodeling of the ECM within the peritoneal space. In response to ongoing inflammatory stimuli, various immune cells, such as lymphocytes, macrophages, and plasma cells, infiltrate the peritoneal tissue, thereby fostering a chronic inflammatory environment that promotes fibrosis. Notably, instances of IgG4-related sclerosing encapsulating peritonitis underscore the role of lymphoplasmacytic inflammation, marked by a significant presence of IgG4-positive plasma cells, which suggests an immune-mediated aspect in the etiology of ACS (37).

Activated immune cells release an array of cytokines and growth factors, including TGF-*β*, interleukins, and tumor necrosis factor-alpha (TNF-*α*), which collectively enhance fibroblast activation and ECM synthesis. Simultaneously, matrix metalloproteinases (MMPs) and their tissue inhibitors play crucial roles in regulating ECM turnover and remodeling. An imbalance in this regulatory mechanism can lead to excessive accumulation of collagen and other matrix proteins, culminating in the formation of the dense fibrous membrane that is characteristic of the abdominal cocoon (65, 72). Moreover, inflammation mediated by immune cells can induce adhesions between bowel loops and the peritoneum, further aggravating mechanical obstruction.

The interaction between immune activation and ECM remodeling is further complicated by the presence of ductal remnants and epithelial structures within the fibrous membrane, as observed in rare pediatric cases. This indicates the possibility of abnormal tissue repair mechanisms and cellular diversity contributing to cocoon formation (35). A comprehensive understanding of these cellular and molecular processes is essential for the development of targeted therapies aimed at modulating immune responses and ECM remodeling, thereby preventing or treating ACS. Furthermore, this mechanistic understanding provides a foundation for the rationale behind immunomodulatory and anti-fibrotic strategies that may enhance surgical management, potentially leading to improved outcomes for patients afflicted by this rare and challenging condition.

### 5.3 Clinical significance of pathological diagnosis

The pathological diagnosis of ACS is of utmost significance in the clinical management of this condition, as it serves as the definitive gold standard for disease confirmation, particularly in intricate or unclear cases. Although imaging techniques, such as contrast-enhanced computed tomography, can indicate the diagnosis by highlighting distinctive characteristics like the fibrocollagenous membrane surrounding the small intestine, these imaging findings frequently fall short of providing a definitive diagnosis due to the potential for overlapping clinical presentations with other causes of intestinal obstruction. A thorough pathological examination of the resected membrane and affected tissues allows for direct visualization of the fibrocollagenous encapsulation and any related inflammatory alterations, thereby unequivocally confirming the diagnosis of ACS. This aspect is especially crucial for distinguishing tuberculous abdominal cocoon from non-tuberculous origins, as the treatment strategies and prognoses vary considerably. Histopathological analysis of tuberculous abdominal cocoon typically reveals granulomatous inflammation accompanied by caseating necrosis, which necessitates the initiation of antitubercular therapy in conjunction with surgical intervention. Conversely, non-tuberculous ACS generally presents with dense fibrocollagenous tissue infiltrated by chronic inflammatory cells but lacks the granulomatous characteristics, thus directing clinicians towards alternative postoperative management approaches. Furthermore, a pathological diagnosis is instrumental in recognizing secondary complications, such as appendiceal involvement resulting from chronic inflammation within the cocoon, which can significantly influence intraoperative decision-making and mitigate the risk of unnecessary extensive resections. For example, a documented case involving a young male patient with ACS illustrated that acknowledging the appendicular involvement as a consequence of chronic inflammation associated with the cocoon facilitated a straightforward appendectomy instead of a more radical hemicolectomy, thereby minimizing surgical morbidity and preserving bowel function (55). Consequently, the role of pathological diagnosis extends beyond mere confirmation of ACS; it also provides critical insights into the etiology and associated pathological alterations, thereby enabling tailored surgical and medical interventions that enhance patient outcomes. In conclusion, the clinical relevance of pathological diagnosis in ACS is rooted in its essential function in confirming the disease, differentiating between tuberculous and non-tuberculous forms, guiding appropriate treatment strategies, and preventing overtreatment in complex cases.

## 6 Advances in treatment strategies and surgical techniques

### 6.1 Indications and efficacy of conservative treatment

Conservative management is typically regarded as the first-line approach for individuals experiencing mild intestinal obstruction due to ACS. This method generally entails bowel rest achieved through fasting, gastrointestinal decompression implemented via nasogastric or intestinal tubes, alongside supportive care that encompasses fluid and electrolyte management. The underlying principle of this treatment modality is to alleviate symptoms and mitigate intestinal distension without the need for invasive procedures. For example, in scenarios where a preoperative diagnosis is established and the obstruction is either incomplete or intermittent, conservative management may be prioritized to minimize surgical risks, particularly in patients with significant comorbidities or those deemed poor candidates for surgery (73). Nonetheless, the efficacy of conservative treatment is frequently restricted to symptomatic relief rather than rectifying the fundamental pathology. The fibrous membrane encasing the small bowel loops, a hallmark of abdominal cocoon syndrome, remains intact despite conservative interventions, rendering this approach predominantly palliative. As a result, patients often face recurrent obstruction episodes even after initial symptom alleviation (44). The chronic inflammatory process responsible for membrane formation and bowel encapsulation persists, which elucidates the challenges in achieving a definitive resolution without surgical intervention. Furthermore, in instances of EPS resulting from PD, conservative management may involve the discontinuation of PD and nutritional support; however, these strategies alone seldom reverse established fibrotic alterations (29). Imaging techniques, such as CT scans, can play a role in tracking disease progression during conservative treatment; however, the persistence or exacerbation of obstructive symptoms frequently necessitates surgical intervention. In conclusion, although conservative treatment is suitable for mild cases and may offer temporary symptom relief, it is generally inadequate for curing ACS. Surgical excision of the fibrous membrane is the definitive approach to restoring bowel motility and preventing recurrent obstruction. Thus, meticulous patient selection and vigilant clinical monitoring are crucial when considering conservative management, with a readiness to transition to surgical intervention if symptoms persist or deteriorate.

### 6.2 Indications and principles of surgical treatment

Surgical intervention for ACS is primarily warranted in the presence of unequivocal signs of intestinal obstruction, bowel ischemia, or perforation—conditions that represent immediate risks to patient survival and necessitate prompt intervention. The existing literature consistently underscores that surgical intervention is essential when conservative treatment proves ineffective or when complications such as complete bowel obstruction or intestinal necrosis arise. For instance, in various case studies and reviews, patients exhibiting severe obstruction or ischemia underwent exploratory laparotomy, which not only confirmed the diagnosis but also facilitated definitive surgical treatment (63, 73). The presence of clinical indicators such as persistent abdominal pain, vomiting, and radiological findings of obstruction or ischemia on CT scans are pivotal in determining the necessity for surgical intervention (12, 42). Additionally, the decision to proceed with surgery is further substantiated when imaging reveals encapsulation of bowel loops accompanied by mesenteric congestion or dilatation, or when clinical signs of deterioration indicate potential bowel compromise (18).

**Figure 2:**
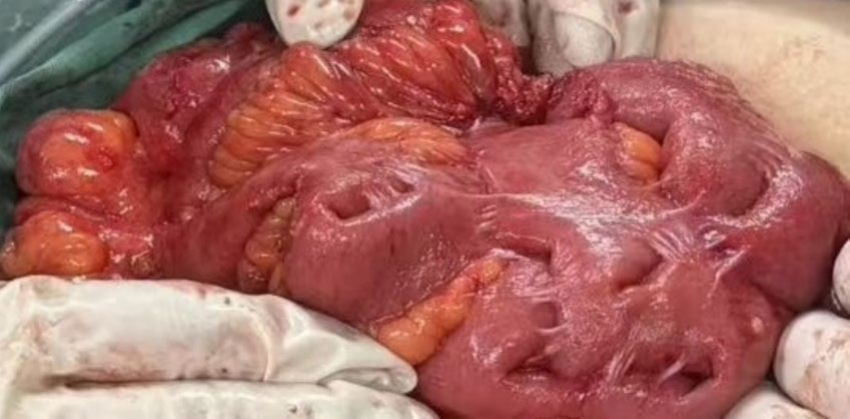
Intraoperative images of patients with abdominal cocoon sign show that the intestinal tubes are in a clustered and adherent state.

The fundamental principles of surgical intervention encompass the thorough excision of the fibrous cocoon membrane alongside careful adhesiolysis aimed at liberating the entrapped intestinal loops. This fibrous membrane, which is primarily composed of fibro-collagenous tissue, functions as a constricting sac encasing the intestines, consequently resulting in either recurrent or acute obstructions. The surgical elimination of this membrane is imperative for the restoration of bowel motility and the prevention of further obstructions (73, 74). Adhesiolysis must be executed with precision to mitigate the risk of iatrogenic bowel injury, a considerable concern due to the presence of dense adhesions linking the bowel to the fibrous membrane (19, 26). The involvement of seasoned surgeons is vital for the safe management of these adhesions, as accidental enterotomies can lead to complications during recovery and may require bowel resection (75). In certain instances, bowel resection accompanied by anastomosis becomes necessary only when irreversible ischemia or gangrene is detected during the surgical procedure (56). The existing literature emphasizes that bowel resection should be restricted to these specific circumstances to conserve as much functional intestine as possible, considering the potential risks associated with short bowel syndrome and other related complications. We present one case of intraoperative images of a patient, showing the intestinal tubes in a mass of adhesions, as shown in Figure 2.

The surgical results documented in existing literature tend to be positive when the established principles are followed diligently. Patients who undergo comprehensive membrane excision and adhesiolysis typically experience uncomplicated postoperative recoveries, marked by the alleviation of obstructive symptoms and a minimal incidence of complications (18, 63). Nevertheless, the intricacy of the surgical procedure escalates in scenarios involving extensive adhesions, concurrent fistulas, or a history of previous abdominal surgeries, which can lead to more intricate dissections and an elevated risk of complications (76). In exceptional and severe cases, particularly those complicated by intestinal necrosis or the formation of fistulas, employing staged surgical strategies alongside meticulous postoperative management, which encompasses drainage and nutritional support, is essential for achieving favorable outcomes (75).

Preoperative imaging techniques, particularly contrast-enhanced CT, are crucial for effective surgical planning, as they facilitate the identification of the extent of bowel involvement and the detection of complications such as ischemia or perforation (77). Despite significant advancements in imaging technology, numerous cases are often diagnosed during surgery due to the ambiguous clinical manifestations and the infrequency of the condition (63, 73). Consequently, maintaining a high level of suspicion and being prepared to undertake surgical intervention when necessary are vital.

In conclusion, the criteria for surgical intervention in cases of ACS are clearly established, particularly in the presence of intestinal obstruction, ischemia, or perforation. The surgical strategy primarily involves the complete removal of the fibrous membrane and meticulous adhesiolysis to liberate the bowel, with bowel resection being reserved for segments deemed nonviable. The successful management of this condition relies on prompt recognition, accurate imaging, and surgical proficiency to reduce complications and enhance patient outcomes (56).

### 6.3 Application and challenges of minimally invasive surgery

Minimally invasive surgical techniques, particularly those involving laparoscopy, have seen a rising application in the treatment of ACS, particularly in cases that are in their early stages and exhibit less extensive fibrous encapsulation. The benefits associated with laparoscopic surgery include diminished surgical trauma, expedited postoperative recovery, and reduced duration of hospital stays, all of which are critical advantages for patients affected by this rare and frequently debilitating disorder. For instance, a documented case illustrated the successful laparoscopic intervention for an abdominal cocoon that was incidentally identified during a surgical procedure for cryptorchidism and seminoma. In this instance, meticulous enterolysis and membrane excision were executed, resulting in a favorable postoperative recovery (78). In another report, the effectiveness of diagnostic laparoscopy was emphasized, not only for confirming the diagnosis but also for enabling adhesiolysis and membrane excision, which culminated in symptom alleviation and positive long-term outcomes (72). These observations highlight the increasing significance of minimally invasive surgical strategies in appropriately selected patients, especially when preoperative imaging indicates minimal fibrous involvement.

Nonetheless, the utilization of laparoscopic surgery in the context of ACS presents notable obstacles (79). A defining characteristic of this condition is the presence of a thick, fibrous membrane surrounding the small intestine, frequently associated with substantial adhesions between the intestinal loops and the peritoneal cavity. In instances where the fibrous membrane exhibits considerable thickness and the adhesions are pronounced, the likelihood of intraoperative bowel injury significantly escalates. Therefore, it is imperative to conduct careful and precise dissection to prevent unintentional enterotomies, which may result in postoperative complications such as enterocutaneous fistulas or peritonitis. For example, in a cohort of 15 patients who underwent surgical intervention for ACS, the importance of meticulous adhesiolysis was underscored as essential for minimizing morbidity. Despite the application of careful surgical techniques, one patient experienced the development of an enterocutaneous fistula necessitating reoperation (69). This underscores the critical need to achieve a balance between sufficient membrane excision and the preservation of bowel integrity.

Additionally, the presence of dense adhesions and increased membrane thickness can extend the duration of surgery and elevate the complexity of the procedure, occasionally leading to a necessary transition from laparoscopic techniques to open laparotomy. In certain documented instances, the diagnosis of ACS was established only during the surgical procedure, where the degree of fibrous encapsulation was so pronounced that open surgery was favored to facilitate safe dissection and complete removal of the membrane (80) Consequently, the surgical approach should be tailored to the individual patient, taking into consideration the severity of the disease, the surgeon’s expertise, and the findings observed during the operation. Preoperative imaging techniques, such as contrast-enhanced computed tomography, can be instrumental in evaluating the level of encapsulation and adhesions, thereby potentially influencing the selection of the surgical method (41).

In conclusion, although laparoscopic surgery presents considerable advantages in managing abdominal cocoon syndrome, its effectiveness is constrained by the severity of fibrous membrane development and adhesions. Cases in the early stages with limited involvement are optimal candidates for minimally invasive strategies, while more advanced cases necessitate careful open surgery to reduce the risk of complications. The cornerstone of successful surgical intervention resides in meticulous patient selection, comprehensive preoperative assessment, and precise intraoperative technique to ensure an effective balance between membrane excision and the preservation of bowel function (66).

### 6.4 Surgical complications and prevention

Surgical management of ACS presents considerable challenges due to the presence of a dense fibrous encapsulation surrounding the intestines, which increases the risk of significant intraoperative and postoperative complications. The predominant surgical complications associated with this condition include intestinal perforation, hemorrhage, and the formation of postoperative adhesions, all of which can severely impact patient outcomes. Intestinal perforation poses a substantial risk during adhesiolysis, as the bowel wall is often fragile and tightly adhered to the fibrous membrane, heightening the likelihood of unintentional injury. For instance, one case report highlighted an instance of intestinal rupture occurring at two ileal locations during surgical intervention for abdominal cocoon, which necessitated resection and anastomosis, followed by intricate postoperative management that included a secondary laparotomy for fistula repair (75). Such complications emphasize the necessity for meticulous surgical techniques and heightened intraoperative awareness. Hemorrhage may occur as a result of extensive dissection involving the vascularized fibrous membrane and adhesions, necessitating careful hemostatic measures to avert significant blood loss. Moreover, postoperative adhesions are a common consequence, potentially resulting in recurrent bowel obstruction or stoma occlusion, as evidenced by cases requiring multiple surgeries to address complications related to adhesions and stoma dysfunction (12). The prevention of these complications is heavily dependent on comprehensive preoperative assessment and meticulous surgical planning. Contrast-enhanced CT is instrumental in defining the extent of bowel encapsulation and ischemic alterations, allowing surgeons to anticipate potential technical challenges and customize their surgical approach accordingly (56, 77). During surgical procedures, it is imperative to perform adhesiolysis with care and precision, ensuring that anatomical planes are accurately identified to mitigate the risk of intestinal damage. The incorporation of minimally invasive approaches, particularly robot-assisted surgery, has been highlighted as beneficial for achieving meticulous dissection in intricate anatomical situations, including those complicated by situs inversus totalis, thus lowering the likelihood of complications (81). Furthermore, supplementary strategies such as the utilization of bioresorbable anti-adhesion membranes and postoperative corticosteroid treatment have demonstrated potential in averting the recurrence of adhesions and fibrosis, as evidenced by veterinary studies on sclerosing encapsulating peritonitis (82). Postoperative protocols should encompass diligent observation for early indicators of complications, such as alterations in drainage fluid that may suggest fistula development, which could require immediate action (75). Ultimately, minimizing surgical risks associated with ACS necessitates a collaborative approach that integrates thorough preoperative imaging, surgical proficiency, and extensive postoperative care. Surgeons should be equipped to address the potential need for bowel resection in instances of ischemia or necrosis while aiming to retain as much functional intestine as possible to avert the onset of short bowel syndrome (56, 57). In conclusion, acknowledging the risk of severe complications and applying preventive measures through meticulous evaluation and surgical methodology are vital for enhancing patient outcomes in those undergoing surgical intervention for ACS.

## 7 Prognostic evaluation and future research directions

### 7.1 Analysis of prognostic factors

The early identification and prompt surgical intervention are pivotal factors that markedly enhance the prognosis for individuals diagnosed with ACS. This medical condition is identified by the fibrous encasement of the small intestine, which results in clinical manifestations including bowel obstruction, abdominal discomfort, and emesis (55). Due to the infrequency and vague clinical features associated with SEP, there is a tendency for delayed or misdiagnosis, potentially leading to increased morbidity and mortality rates. Imaging techniques, especially CT, are crucial in heightening diagnostic suspicion and facilitating the diagnostic process; nevertheless, a conclusive diagnosis typically necessitates an exploratory laparotomy. Surgical intervention, which entails the removal of the fibro-collagenous membrane and adhesiolysis, remains the primary treatment approach for symptomatic presentations. Prompt surgical procedures not only alleviate obstruction but also avert the development of severe complications such as bowel ischemia or perforation. Evidence suggests that patients receiving early surgical treatment generally achieve better outcomes and lower mortality rates, highlighting the critical nature of swift recognition and intervention in cases of ACS (20).

In contrast, the clinical landscape of tuberculous abdominal cocoon poses considerable challenges, characterized by an elevated likelihood of recurrence and increased mortality rates. Tuberculosis-related ACS represents a variant of secondary sclerosing peritonitis, wherein the infiltration of *Mycobacterium tuberculosis* into the peritoneum results in significant fibrotic encapsulation and sustained inflammatory responses. This particular etiology is linked to a more severe disease trajectory and a less favorable prognosis when compared to idiopathic or alternative secondary types. Diagnosing tuberculous ACS is frequently hindered by the presence of nonspecific clinical manifestations, necessitating microbiological or histopathological verification for accurate identification. Furthermore, the inflammatory environment and immune reactions associated with tuberculosis may render patients susceptible to recurrent fibrotic encapsulation, even following surgical intervention. Therefore, these individuals necessitate not only surgical procedures to alleviate obstruction but also extended anti-tubercular treatment to manage the underlying infection effectively. Despite implementing these strategies, the potential for relapse and mortality remains pronounced, underscoring the critical need for diligent postoperative surveillance and comprehensive therapeutic approaches specifically designed for the tuberculous origin (33). Collectively, this evidence accentuates that while timely diagnosis and surgical intervention can generally enhance outcomes in abdominal cocoon syndrome, the presence of tuberculosis significantly alters the prognosis, necessitating a multidisciplinary strategy to optimize patient survival and minimize recurrence rates (69).

### 7.2 Long-term follow-up and quality of life

Long-term monitoring is crucial for individuals who have undergone surgical intervention for ACS, in order to evaluate intestinal functionality and identify any potential recurrence. Given the propensity of ACS to result in acute intestinal obstruction, postoperative observation is primarily concerned with examining bowel motility, assessing nutritional status, and recognizing early indications of adhesion reforming or fibrotic encapsulation that could jeopardize intestinal patency. A notable case involving a 10-year-old female patient with secondary ACS following abdominal surgery underscores the significance of prompt and precise postoperative care, along with consistent follow-up appointments, to secure favorable long-term results (33). Such follow-up generally encompasses clinical assessments, imaging modalities aimed at evaluating the integrity and functionality of the intestines, and laboratory analyses to track nutritional indicators and levels of inflammation. The early identification of complications, including recurrent obstruction or infection, facilitates timely intervention, which is essential in mitigating further morbidity.

The quality of life (QoL) in individuals who have undergone surgery for SEP is markedly affected by the extent of intestinal functional impairment and the occurrence of surgical complications. Intestinal dysfunction may present as persistent abdominal discomfort, changes in bowel patterns, malabsorption issues, or nutritional deficiencies, all of which can significantly hinder daily activities and overall well-being. Furthermore, complications arising from surgery, including adhesions, anastomotic strictures, or infections, may extend the recovery period and contribute to ongoing symptoms. In pediatric populations, these issues can adversely affect growth and development, thereby further influencing QoL. Consequently, comprehensive postoperative management should not only focus on the preservation of intestinal function but also encompass psychosocial dimensions, nutritional rehabilitation, and physical recovery. A multidisciplinary approach that integrates the expertise of surgeons, gastroenterologists, nutritionists, and psychologists is often essential to optimize long-term results and improve the quality of life for patients. The positive prognosis observed in pediatric cases highlights that with precise diagnosis, suitable perioperative care, and thorough follow-up, patients can attain significant functional recovery and sustain a satisfactory quality of life (33).

### 7.3 Future research priorities

Although ACS has garnered increasing attention, its exact pathophysiological mechanisms remain inadequately understood. Existing literature indicates that chronic inflammation of the peritoneum contributes to the development of fibro-collagenous membranes that envelop the small intestine; however, the specific molecular and cellular pathways that facilitate this fibrotic phenomenon have not been thoroughly characterized. Idiopathic ACS is characterized by the absence of identifiable precipitating factors, while secondary forms are linked to conditions such as tuberculosis, neoplasms, or previous abdominal surgeries, suggesting that various etiological factors may converge upon a shared fibrotic pathway (83). Future investigations should aim to elucidate the molecular signaling pathways implicated in peritoneal fibrosis, with particular emphasis on the roles of inflammatory cytokines, fibroblast activation, extracellular matrix accumulation, and the infiltration of immune cells. For example, research into the involvement of TGF-*β*, matrix metalloproteinases, and profibrotic mediators could provide insights into the fibrogenic environment. Furthermore, pinpointing specific molecular targets may facilitate the creation of targeted therapies designed to inhibit or reverse membrane formation, potentially decreasing the necessity for invasive surgical interventions. Given the infrequency of AC, collaborative efforts across multiple centers and the establishment of patient registries could enhance the collection of biological samples for molecular studies. Additionally, animal models that replicate the fibrotic encapsulation could serve as valuable platforms for the preclinical evaluation of antifibrotic treatments. Ultimately, a deeper comprehension of the pathogenesis will enable the development of personalized molecular therapies, thereby improving patient outcomes and diminishing the morbidity associated with ACS.

The preoperative identification of ACS presents significant difficulties, primarily due to its nonspecific clinical manifestations and the infrequency of the condition. Typically, diagnoses are established during surgical procedures, particularly in emergency situations involving intestinal obstruction (63). Nevertheless, advancements in imaging techniques, especially contrast-enhanced CT, have shown promise in revealing distinctive characteristics associated with abdominal cocoon syndrome, including the presence of clustered small bowel loops enveloped by a dense fibro-collagenous membrane, thickening of the peritoneum, and the occurrence of loculated ascites (50, 55). In spite of these advancements, the sensitivity and specificity of existing imaging protocols remain inadequate, and radiologists may not be sufficiently informed about this syndrome due to its uncommon nature. Future investigations should focus on enhancing imaging criteria and formulating standardized diagnostic algorithms that integrate CT, MRI, and potentially positron emission tomography (PET) to improve detection precision. Cutting-edge imaging modalities, such as diffusion-weighted MRI, could offer further insights into tissue characteristics and inflammatory processes. Additionally, the application of artificial intelligence and machine learning techniques may be beneficial in aiding pattern recognition and differentiating this syndrome from other etiologies of intestinal obstruction. Furthermore, the correlation of imaging results with histopathological and clinical data will bolster diagnostic accuracy. Achieving an early and precise preoperative diagnosis will enable timely surgical preparation, minimize intraoperative complications, and enhance patient outcomes.

The primary treatment for symptomatic ACS, particularly in instances of acute or recurrent intestinal obstruction, continues to be surgical excision of the fibrous membrane along with adhesiolysis (63). Nonetheless, the surgical procedure frequently encounters challenges due to the presence of dense adhesions and fragile bowel tissue, which heightens the likelihood of iatrogenic enterotomies and increases postoperative complications (84). The introduction of laparoscopic techniques has demonstrated potential benefits in selected cases, such as decreased postoperative pain, reduced length of hospital stay, and expedited recovery (66). However, the application of laparoscopic surgery in abdominal cocoon syndrome necessitates a high level of surgical skill and may face limitations due to extensive fibrosis and bowel involvement. Future investigations should concentrate on enhancing minimally invasive methodologies, including the refinement of patient selection criteria, the development of specialized instruments for safe adhesiolysis, and the incorporation of intraoperative imaging or navigation technologies to mitigate complications. Furthermore, the exploration of adjunctive therapies, such as anti-inflammatory or antifibrotic pharmacological agents administered during the perioperative period, may help diminish membrane formation and recurrence of adhesions. Innovative strategies, including intraperitoneal drug delivery systems or targeted molecular therapies, could serve as complementary measures to surgical intervention and enhance long-term patient outcomes. Additionally, it is imperative to establish enhanced recovery protocols specifically designed for patients with abdominal cocoon syndrome, aimed at reducing surgical stress and promoting healing. Collectively, these advancements are intended to minimize surgical risks and improve the quality of life for individuals affected by this rare yet complex condition.

## 8 Conclusion

In conclusion, ACS, though rare, represents a clinically significant cause of intestinal obstruction with a multifaceted etiology, among which tuberculosis infection stands out as a primary precipitating factor. From an expert perspective, the complexity of its pathogenesis underscores the necessity of a nuanced understanding that integrates infectious, immunological, and possibly idiopathic mechanisms. This comprehensive approach facilitates a balanced interpretation of the diverse research findings, which often reflect varying regional prevalence and diagnostic challenges.

Clinically, the heterogeneous presentation of ACS demands a high index of suspicion. Imaging modalities such as CT have markedly improved preoperative diagnostic accuracy, yet definitive diagnosis still hinges on intraoperative findings and histopathological confirmation. This diagnostic paradigm highlights the importance of combining radiological expertise with surgical and pathological insights to optimize patient outcomes.

Surgical intervention remains the cornerstone of treatment, with the advent of minimally invasive techniques offering promising avenues for reducing operative morbidity and enhancing recovery. However, the potential for intraoperative complications necessitates meticulous surgical planning and expertise. Balancing the benefits of novel surgical approaches with their inherent risks is critical, and ongoing refinement of these techniques will likely shape future standards of care.

Early diagnosis coupled with individualized therapeutic strategies emerges as pivotal in improving prognosis. Tailoring treatment plans to the patient’s specific etiological factors, disease severity, and comorbid conditions aligns with contemporary precision medicine principles and holds the potential to mitigate complications and recurrence.

Looking ahead, research efforts should prioritize elucidating the underlying pathogenic mechanisms, particularly the role of tuberculosis and other infectious agents, to inform targeted preventive and therapeutic interventions. Moreover, innovation in diagnostic modalities and surgical technologies will be instrumental in advancing clinical management. Collaborative multidisciplinary research and clinical trials are essential to harmonize diverse perspectives and translate emerging evidence into practice.

In summary, ACS exemplifies a complex clinical entity where expert integration of epidemiological, diagnostic, and therapeutic knowledge is vital. By balancing differing research viewpoints and embracing technological advancements, the medical community can enhance patient care and foster meaningful progress in understanding and managing this challenging condition.
